# We need to correct the wide-spread omission of equal contribution in article indexing

**DOI:** 10.1371/journal.pbio.3003746

**Published:** 2026-04-20

**Authors:** Wenying Shou

**Affiliations:** Department of Genetics, Evolution and Environment, Centre for Life’s Origins and Evolution, University College London, London, United Kingdom

## Abstract

Equal contribution designations (co-first and co-last authorship) is on the rise, yet this information is routinely lost, creating inequity in recognition and crediting. This Perspective calls for improvements to the system for transferring this information to indexing sites such as PubMed.

## Teaser

As team science grows, so do ‘equal contribution’ designations, yet this information is routinely hidden or lost, creating inequity in recognition and crediting. We must fix this problem, now.

The increasing use of ‘equal contribution’ designations (i.e., co-first or co-last authors) [1] rightfully reflects the rising importance of collaborative and interdisciplinary science. In some biomedical and clinical journals, co-first authorship was absent in 1990, but appeared in >30% of research articles by 2012 [2]. This shift reflects the evolving nature of modern science. However, our system for credit and recognition has not kept pace.

Author contributions remain the central currency of academic careers. Initiatives such as CRediT (Contributor Roles Taxonomy) [3] have advanced transparency by clarifying author roles. Yet, these detailed statements often remain unread by time-pressed evaluators who rely instead on simple heuristics: first authors are seen as drivers of projects, while last authors are seen as team leaders and supervisors. Consequently, middle authors receive diminishing credit, a problem that has fueled the rise of the equal contribution designation.

Acknowledging this problem invites a philosophical critique: is focusing on ‘first’ and ‘last’ authors itself antithetical to team science? Some fields, notably mathematics and economics, have historically employed alphabetical authorship to sidestep the politics of ordering. However, use of the alphabetical authorship system is on the decline as the average number of coauthors increases [4], and carries its own biases. For example, in economics, researchers with earlier surname initials are significantly more likely to receive tenure at top departments and become fellows of prestigious societies; a phenomenon that is absent in psychology, where contribution-based ordering is the norm [5]. Others have argued that we should entirely move away from a system where author position dictates credit [6]. However, positional hierarchy remains the operational currency of academic career advancement, and an incomplete transition can do more harm than good [7]. Thus, for most of the life sciences and beyond, contribution-based author order is the entrenched, if imperfect, language of credit.

Currently, most major publishers include equal contribution notes in the article PDF. However, the information about equal contribution is often lost after publication. Major databases such as PubMed, ORCID, Google Scholar, and ResearchGate display only the raw author list. As downstream systems increasingly depend on these records for metrics and evaluations, this omission triggers a silent distortion of career credit and visibility. For example, “X & Y et al.” becomes “X et al.”. As a result, seminar invitations become preferentially channeled to the first and last authors, and not to the co-first or co-last authors. I have witnessed presentations where the first-listed co-first author took the podium, yet their slides—featuring citations often pulled directly from PubMed—failed to acknowledge the equal contribution, much to the demoralization of their coauthor.

The problem of equal authors not being credited equally was highlighted more than a decade ago [2], yet it remains unresolved. This inequity is exacerbated by the growing emphasis on quantitative metrics, many of which explicitly reward position-based credit [8,9]. For example, GScholarLens, a new bibliometric tool, automatically assigns value based on list position, granting a last author 100% credit, a first author 90% credit, and a second author only 50% credit [10]. Without metadata that reflects equal contributions, these metrics will systematically disadvantage many scientists who share equal responsibility for a study’s success.

In the short term, major academic profiling platforms such as ORCID and Research Gate should develop and promote clear features that allow authors to self-report equal contributions, or even better, list the actual contributions. Presently, only Google Scholar offers a rudimentary manual option that allows author names to be annotated (for instance, with symbols such as asterisks).

**Fig 1:**
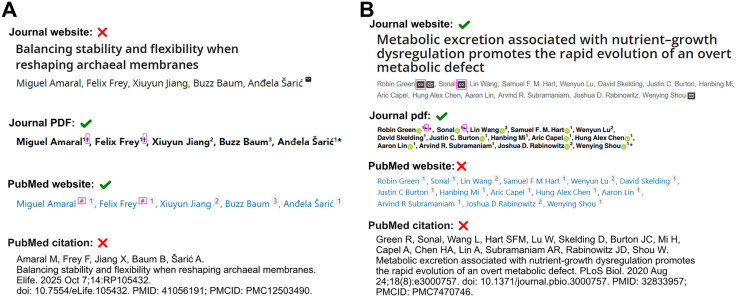
Loss of equal contribution information despite adherence to best practices in JATS XML. Two representative articles (A [11] and B [12]) illustrate how equal contribution designations (highlighted in magenta boxes) are preserved in journal PDFs but systematically absent from PubMed citations (last row). This loss is reflected in the final two citations of this article.

For a more long-term solution, the system used to denote author contributions will need to have expanded functionality, and be standardized and widely adopted. First, there needs to be more functionality in the Journal Article Tag Suite (JATS) XML standard. For example, in JATS, ‘@equal-contrib=“yes”’ is used to mark equally contributing authors, but it can only be used for one set of authors. As a sticking plaster for this limitation, JATS4R advises publishers to always include a clarifying footnote, but this content is not machine readable. Perhaps because of this, PubMed only allows publishers to submit ‘@equal-contrib’ flags, but not any clarifying footnotes. Due to this constraint—and other factors yet to be identified—information on equally contributing authors is commonly lost, even when journals follow best practice (Fig 1).

Second, the lack of standardization needs to be rectified. For example, while one publisher may use the attribute ‘@equal-contrib=“yes”’, a different publisher could use the ‘specific-use’ attribute, which is a free-text field. Yet other publishers are stuck using legacy systems that output only footnotes.

And finally, publishers would be strongly incentivized to comply if implementation were made a pre-requisite for journal indexing in major databases. Thus, prominent indexing entities such as PubMed, Web of Science, and Scopus are uniquely positioned to drive this change. They could create a ‘compliance checkpoint’ by requiring adherence to reporting standards. For publishers, the prospect of losing indexing privileges poses an existential threat, making this one of the most viable levers for enforcement.

As the pace of team science accelerates, we can no longer afford to allow digital carelessness to undermine scientific fairness. The time for this critical reform is now.
